# Diagnostic rate of germline pathogenic variants in pancreatic ductal adenocarcinoma patients using whole genome sequencing

**DOI:** 10.3389/fgene.2023.1172365

**Published:** 2023-05-10

**Authors:** An-Ko Chung, Ro-Ting Lin, Chun-Chieh Yeh, Chi-Ying Yang, Chang-Jiun Wu, Pei-Lung Chen, Jaw-Town Lin

**Affiliations:** ^1^ Graduate Institute of Medical Genomics and Proteomics, National Taiwan University, Taipei, Taiwan; ^2^ Department of Internal Medicine, National Taiwan University College of Medicine, Taipei, Taiwan; ^3^ Department of Occupational Safety and Health, College of Public Health, China Medical University, Taichung, Taiwan; ^4^ School of Medicine, China Medical University, Taichung, Taiwan; ^5^ Department of Surgery, China Medical University Hospital, Taichung, Taiwan; ^6^ Department of Internal Medicine, Digestive Medicine Center, China Medical University Hospital, Taichung, Taiwan; ^7^ Department of Genomic Medicine, MD Anderson Cancer Center, University of Texas, Houston, TX, United States; ^8^ Department of Medical Genetics, National Taiwan University Hospital, Taipei, Taiwan; ^9^ Division of Gastroenterology and Hepatology, Department of Internal Medicine, E-Da Hospital, Kaohsiung, Taiwan

**Keywords:** pancreatic ductal adenocarcinoma (PADC), whole genome sequencing (WGS), germline genetic testing, structural variant (SV), cancer genetic

## Abstract

Identification of germline pathogenic variants in cancer patients is critical for treatment planning, genetic counseling, and health policymaking. However, previous estimates of the prevalence of germline etiology of pancreatic ductal adenocarcinoma (PDAC) were biased because they were based only on sequencing data of protein-coding regions of known PDAC candidate genes. To determine the percentage of patients with PDAC carrying germline pathogenic variants, we enrolled the inpatients from the digestive health clinics, hematology and oncology clinics, and surgical clinics of a single tertiary medical center in Taiwan for whole genome sequencing (WGS) analysis of genomic DNA. The virtual gene panel of 750 genes comprised PDAC candidate genes and those listed in the COSMIC Cancer Gene Census. The genetic variant types under investigation included single nucleotide substitutions, small indels, structural variants, and mobile element insertions (MEIs). In 8 of 24 (33.3%) patients with PDAC, we identified pathogenic/likely pathogenic variants, including single nucleotide substitutions and small indels in *ATM*, *BRCA1*, *BRCA2*, *POLQ*, *SPINK1* and *CASP8*, as well as structural variants in *CDC25C* and *USP44*. We identified additional patients carrying variants that could potentially affect splicing. This cohort study demonstrates that an extensive analysis of the abundant information yielded by the WGS approach can uncover many pathogenic variants that could be missed by traditional panel-based or whole exome sequencing-based approaches. The percentage of patients with PDAC carrying germline variants might be much higher than previously expected.

## Introduction

Pancreatic ductal adenocarcinoma (PDAC) has an extremely poor prognosis. Approximately 10% of unselected patients with PDAC carried germline pathogenic variants (Yurgelun et al., 2019; Gardiner et al., 2022), with the rate being 30% among cases in populations with a strong family history of cancer and/or common founder variants (e.g., in Ashkenazi Jews) (Gardiner et al., 2022). These estimations of germline pathogenic variants related to PDAC might be biased because the number of candidate genes that had their coding regions tested is limited, and the approaches used were panel-based or whole exome sequencing (Yurgelun et al., 2019; Gardiner et al., 2022). This emphasizes a knowledge gap regarding the diagnostic rate of germline pathogenic variants in patients with PDAC using a comprehensive whole genome sequencing (WGS) approach. To ensure the quality of therapeutic planning, early genetic counseling for at-risk relatives, and health policymaking, precise data on the identification of germline pathogenic variants is critical.

## Materials and methods

### Subjects and sample collection

This retrospective cohort study recruited inpatients diagnosed with PDAC (C25.0–C25.9, based on ICD-10) in a tertiary medical center in Taiwan; details are available in our previous publication (Lin et al., 2022). We recruited 24 patients (men: 19; women: 5; median age at diagnosis: 56.7 years). Regarding lesion location, the head of the pancreas (C25.0) was the most common (13 patients), followed by the tail (C25.2; 7 patients), the body (C25.1; 3 patients), and both the head and body (1 patient). The study was approved by the Research Ethics Committee III of the China Medical University and Hospital (CMUH109-REC3-026). Genomic DNA was extracted from the participants’ peripheral blood mononuclear cells.

### WGS and quality analysis

WGS was performed on the Illumina NovaSeq platform (Illumina, San Diego, CA, United States), with 2 × 150 bp paired-end reads to achieve 30 × coverage, followed by an adapter trimming and low-quality bases filtering with Phred quality scores greater than 30.

### Germline variant detection and interpretation

Sequence analysis was conducted based on GATK Best Practice workflow (McKenna et al., 2010) (v4.2). Paired-end reads were aligned to the reference genome (GRCh38/hg38) using BWA-MEM(Li and Durbin, 2009) (v0.7.17). Variant calling was conducted using HaplotypeCaller. Variant quality score recalibrations were performed using VariantRecalibrator. Simple variants (e.g., single nucleotide variants or small indels) were then ready for annotation and automatic interpretation based on five classes (pathogenic, likely pathogenic, uncertain significance, likely benign, and benign) following the American College of Medical Genetics and Genomics guideline (Richards et al., 2015) using TAIGenomics software (https://www.taigenomics.com). Variants with high allele frequencies in the Genome Aggregation Database (gnomAD, https://gnomad.broad institute.org) or Taiwan Biobank (http://taiwanview.Twbiobank.Org.tw/index) databases were filtered out. Annotations of pathogenic and likely pathogenic variants were manually confirmed. Variants identified in the known PDAC genes or known cancer genes in DNA repair pathway (Supplementary Table S1) and the COSMIC Cancer Gene Census (CGC) (https://cancer.sanger.ac.uk/census) were selected (Supplementary Figure S1 **)**. We remove variants which are with read depth ≤10, genotype quality ≤20, or allele balance ≤0.2.

The potential of variants to affect splicing was explored using SpliceAI (Jaganathan et al., 2019). Variants with allele frequencies <1% and located in the candidate genes were tested. A score greater than 0.5 was considered positive.

Mobile element insertions (MEIs) were investigated using SCRAMble (Torene et al., 2020) (v1.0.2) and MELT (Gardner et al., 2017) (v2.2.2). All VCF files from SCRAMble and MELT were annotated using AnnotSV(Geoffroy et al., 2018) (v3.0.9).

Structural variants were examined using a combination of callers including Manta (Chen et al., 2016) (v1.6), Delly (Rausch et al., 2012) (v0.8.7), and SvABA (Wala et al., 2018) (v1.1.0). Structural variants detected by at least two callers were selected using SURVIVOR (Jeffares et al., 2017) (v1.0.7), followed by annotation using AnnotSV(Geoffroy et al., 2018) (v3.0.9).

## Results

Between July 2020 and December 2020, a total of 24 participants fulfilled the inclusion criteria and had all biospecimens available (Supplementary Table S2 **)**.

### Single nucleotide variants and small indels

To identify pathogenic variants, we first constructed a virtual panel of 750 genes, comprising known PDAC genes or known cancer genes in DNA repair pathway (Supplementary Table S1) and the COSMIC CGC Panel. From the list of single nucleotide variants and small indels identified in our cohort, we found six heterozygous pathogenic variants in six different patients. They include DNA damage response and DNA repair genes (*ATM*, *BRCA1*, *BRCA2*, and *POLQ*), pancreatitis gene (*SPINK1*) and cell apoptosis gene (*CASP8*; Table 1).

**TABLE 1:** Disease-causing germline variants or small indels identified in PDAC patients.

Gene	Position	Consequence	Variant classification^a^	Classes	gnomAD v3.1.2		ClinVar clinical significance	RC (Alt/Total)	Sample	Patient risk events
					AF (Total/Eas)	AC (non-cancer)				
*ATM*	11:108335004	c.8046_8047insATACAGTC (p.T2682fs)	Frameshift insertion	LP (PVS1, PM2)				17/28	B0018	Family history of cancer
*BRCA1*	17:43106477	c.190dupT (p.C64fs)	Frameshift insertion	LP (PVS1, PM2)				11/24	B0037	Family history of cancer
*BRCA2*	13:32363178	c.7977-1G>T	Splice acceptor variant	P (PVS1, PM2, PP5)	3.19E-05/6E-04	1	P/LP (Hereditary cancer)	14/27	B0006	Pancreatitis
*POLQ*	3:121519913	c.C1426T (p.Q476X)	Stopgain	LP (PVS1, PM2)				12/20	B0035	
*SPINK1*	5:147828020	c.194 + 2T>C	Splice donor variant	P (PVS1, PM2, PP5)	2E-04/3.2E-03	22	P (9); VUS(1) (Hereditary pancreatitis)	9/15	B0009	Pancreatitis, thyroid cancer
*CASP8*	2:201258276	c.45_46insAACTTCTTCCT (p.R15fs)	Frameshift insertion	LP (PVS1, PM2)	3.19E-05/6E-04	1		14/29	B0014	Pancreatitis

^a^ Variants in the splicing site ( ± 2 bps) were annotated as splice variants.

AC: allele count; AF: allele frequency; Alt: alternative read count; Eas: East Asian; LP: likely pathogenic; P: pathogenic; RC: read count; VUS: uncertain significance.

**FIGURE 1:**
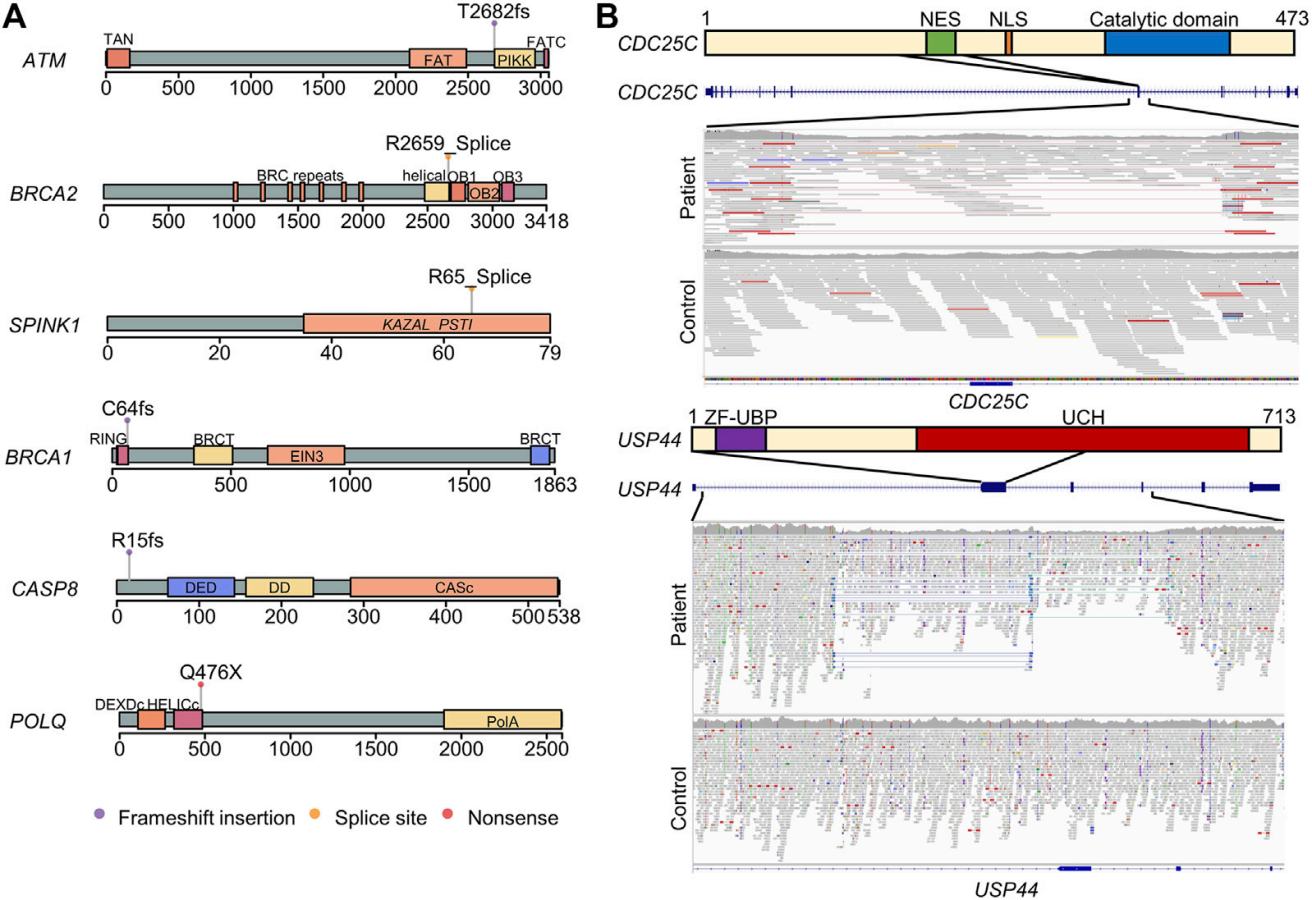
Schematic diagram of the pathogenic germline variants and structural variants identified in patients with pancreatic ductal adenocarcinoma. **(A)** Protein structures and domains were generated using maftools package. The lollipops represent the positions of pathogenic germline variants. **(B)** Deletion in the region containing nuclear export signal in *CDC25C*. The upper panel represented the *CDC25C* protein structure and domain. The middle panel is a screenshot of UCSC Genome Browser in the region of *CDC25C* gene. The lower panel is a screenshot of integrative genomics viewer of the deletion region. The paired reads with unexpected insert-size are visualized in the link line with red color. NES, nuclear export signal. NLS, nuclear localization sequence. A combined deletion and inversion in the second exon of *USP44*. The read depth decreased in the patient compared to the control, and paired-end inversion reads are visualized with light and dark blue colors.

All variants disrupted at least one protein domain in these genes, suggesting that variants lead to loss of function (Figure 1A). Specifically, the patient carrying the *SPINK1* (c.194 + 2T>C) variant had a history of chronic pancreatitis (Table 1). Two variants in *ATM* and *BRCA1* were identified in patients with a family history of cancer (Table 1).

### Variants with potential to affect splicing

We found six heterozygous variants predicted to alter splicing; two in DNA repair-associated genes (*BRCA1* and *BAP1*) and four in tumor suppressor genes (*ARHGEF10L*, *ELL*, *MYH9*, and *NCOR2*) (Supplementary Table S3). Due to a lack of mRNA/cDNA data to confirm the change in splicing patterns, they were not counted as pathogenic variants in this study.

### Structural variants or MEIs

We observed one in-frame deletion of the seventh exon (amino acids 154–205) of *CDC25C*, resulting in the loss of the nuclear export signal (amino acids 190–199) but preserving the catalytic domain and nuclear localization signal; the deletion may cause the accumulation of *CDC25C* in the nucleus and promote a cell cycle without cellular localization control (Figure 1B).

We found a complex structural variant, indicating an inversion and deletion event, interrupting *USP44* (Figure 1B). *USP44* is a recently discovered tumor suppressor gene implicated in PDAC (Yang et al., 2019).

MEIs may interrupt gene function, but we did not find any suspected MEIs in any gene in our PDAC virtual panel.

## Discussion

We identified pathogenic germline variants in eight out of 24 (33.3%) patients with PDAC in Taiwan. This high rate may be partially explained by the WGS approach to examine a comprehensive list of 750 genes, diverse genetic variant types, and sequence information within non-coding regions. Our cohort was not considered to be of younger age (range, 36.1–82.5 years) or have a stronger family history (Lin et al., 2022).

Among the eight variants, the pathogenicity prediction (Richards et al., 2015) and gene-disease correlation of five variants or small indels in *ATM*, *BRCA1*, *BRCA2*, *POLQ*, and *SPINK1* were unequivocal. The pathogenicity predictions of the frameshift insertions in *CASP8* was also convincing. Two of them (*BRCA2* c.7977-1G>T; *SPINK1* c.194 + 2T>C) are listed in the ClinVar database. The *BRCA2* variant was consistently reported to be pathogenic; for the *SPINK1* variant, there were conflicting interpretations of pathogenicity, with nine identifying it as pathogenic and one as uncertain significance. These two variants had both been reported as pathogenic variants in published pancreatic cancer studies (Chian et al., 2021; Yin et al., 2022). Although the other variants in *BRCA1*, *ATM*, and *POLQ* were not previously reported and were identified as novel variants in this current study, pathogenic variants in these genes have also been reported in pancreatic cancer and therefore these genes have been known to cause PDAC (Earl et al., 2020; Mizukami et al., 2020). Inactivation and somatic mutation of *CASP8* are reported in various cancers (Mandal et al., 2020), and a recent report found a colorectal cancer patient with somatic loss of heterozygosity in *CASP8* (Choi et al., 2021). In addition, the level of caspase 8, which is encoded by *CASP8*, was reduced in pancreatic cancer according to a previous study (Jakubowska et al., 2016). The two structural variants were also predicted to have major effects on *CDC25C* and *USP44*, respectively, and while the first *CDC25C* has not been related to PDAC, the latter has recently begun to be linked to PDAC (Yang et al., 2019). Therefore, *CASP8* and *CDC25C* are suggested to be novel pathogenic genes of PDAC, and this study supports the potential of *USP44* to cause PDAC.

We also identified six deep intronic variants predicted to alter splicing in DNA repair-associated genes (*BRCA1* and *BAP1*) or tumor suppressor genes (*ARHGEF10L*, *ELL*, *MYH9*, and *NCOR2*; Supplementary Table S3). They might be disease-causing variants related to PDAC, but we could not confirm this due to a lack of data. We did not identify disease-causing MEIs in our sample.

This study had a modest sample size and lacked confirmatory experiments to determine the disease-causing roles of the identified variants, leaving space for future investigation. Comprehensive and thorough genomic analyses in large cohorts are needed to support our finding that the prevalence of PDAC patients carrying pathogenic germline variants might be higher than previously estimated. Overall, our results demonstrate the potential for the WGS-based approach to uncover pathogenic genes/variants that could be missed by traditional panel-based or WES-based approaches. Although *CASP8*, *CDC25C*, and *USP44* have been undervalued, they were shown to be plausible PDAC genes. The percentage of patients with PDAC carrying germline etiology (33.3% in this study) might be much higher than previously expected.

## Data Availability

The original contributions presented in the study are publicly available. This data can be found here: PRJNA947736.
