# Supplementary material for: Diagnostic rate of germline pathogenic variants in pancreatic ductal adenocarcinoma patients using whole genome sequencing
Source: Front Genet. 2023 May 10;14:1172365. doi: 10.3389/fgene.2023.1172365 (PMC10205989; doi:10.3389/fgene.2023.1172365)

**Supplementary information**

**Supplementary Table 1. List of known pancreatic ductal adenocarcinoma predisposition and cancer genes**

| **Gene** | **Pathway** |
| --- | --- |
| *CDKN2A* | Cell growth |
| *KRAS* | Cell growth |
| *PTEN* | Cell growth |
| *SMAD4* | Cell growth |
| *STK11* | Cell growth |
| *TP53* | Cell growth |
| *CDC25A* | Cell cycle |
| *CDC25C* | Cell cycle |
| *APC* | Cell migration |
| *PALLD* | Cell invasion |
| *ATM* | DNA damage response |
| *CHEK1* | DNA damage response |
| *CHEK2* | DNA damage response |
| *MLH1* | DNA mismatch repair |
| *MSH2* | DNA mismatch repair |
| *MSH6* | DNA mismatch repair |
| *PMS2* | DNA mismatch repair |
| *EPCAM* | Deletion of *EPCAM* silencing *MSH2* expression |
| *BARD1* | DNA repair |
| *BRCA1* | DNA repair |
| *BRCA2* | DNA repair |
| *FANCA* | DNA repair |
| *FANCC* | DNA repair |
| *FANCG* | DNA repair |
| *FANCM* | DNA repair |
| *MUTYH* | DNA repair |
| *NBN* | DNA repair |
| *PALB2* | DNA repair |
| *POLN* | DNA repair |
| *POLQ* | DNA repair |
| *RAD50* | DNA repair |
| *RAD51C* | DNA repair |
| *RAD51D* | DNA repair |
| *CFTR* | Pancreatitis |
| *CPA1* | Pancreatitis |
| *CPB1* | Pancreatitis |
| *CTRC* | Pancreatitis |
| *GGT1* | Pancreatitis |
| *PRSS1* | Pancreatitis |
| *SPINK1* | Pancreatitis |

**Supplementary Table 2. Participants’ demographics**

| **Characteristic** | N = 24 | (%) |
| --- | --- | --- |
| **Age (years)** | |  |
| Median | 56.7 |  |
| Range | 36.1–82.5 |  |
| **Gender** |  |  |
| Female | 5 | 21 |
| Male | 19 | 79 |
| **Race** |  |  |
| Asian | 24 | 100 |

**Supplementary Table 3. SpliceAI-predicted germline variants affecting splicing in the 750-gene pancreatic ductal adenocarcinoma virtual panel identified in patients with pancreatic ductal adenocarcinoma**

| **Gene** | **Position** | **Consequence** | **Variant Classification** | **SpliceAI*** | **GnomAD Allele Frequency (Total / East Asian)** | **ClinVar Clinical Significance** | **Sample** | **Patient risk events** |
| --- | --- | --- | --- | --- | --- | --- | --- | --- |
| *BRCA1* | 17:43067551 | c.5074+57C>T | intron variant | DG 0.74 (2 bp) | 0.0002 / 0.0039 | . | B0004 | Diabetes |
| *ARHGEF10L* | 1:17621858 | c.943-6G>A | splice variant | AG 0.73 (3 bp) | 0.0000638 / 0.0013 | . | B0003 | Diabetes, colon cancer |
| *BAP1* | 3:52407220 | c.C534T:p.G178G | synonymous SNV | DG 0.59 (2 bp) | 0.0003 / 0.0058 | Benign/Likely benign | B0002 | . |
| *ELL* | 19:18465249 | c.469+163G>C | intron variant | DG 0.51 (-13 bp) | 0.00006371 / 0.0013 | . | B0012 | Family history of cancer, diabetes |
| *MYH9* | 22:36299117 | c.2977-75C>T | intron variant | DG 0.90 (2 bp) | . | . | B0031 | Diabetes |
| *NCOR2* | 12:124456930 | c.762+176G>A | intron variant | DG 0.58 (-1 bp) | . | . | B0019 | Family history of cancer, cholelithiasis, diabetes |

*AG: acceptor gain; AL: acceptor loss; DG: donor gain; DL: donor loss.

**Supplementary Figure 1. Flow chart showing the step-wise analysis of the whole genome sequencing**


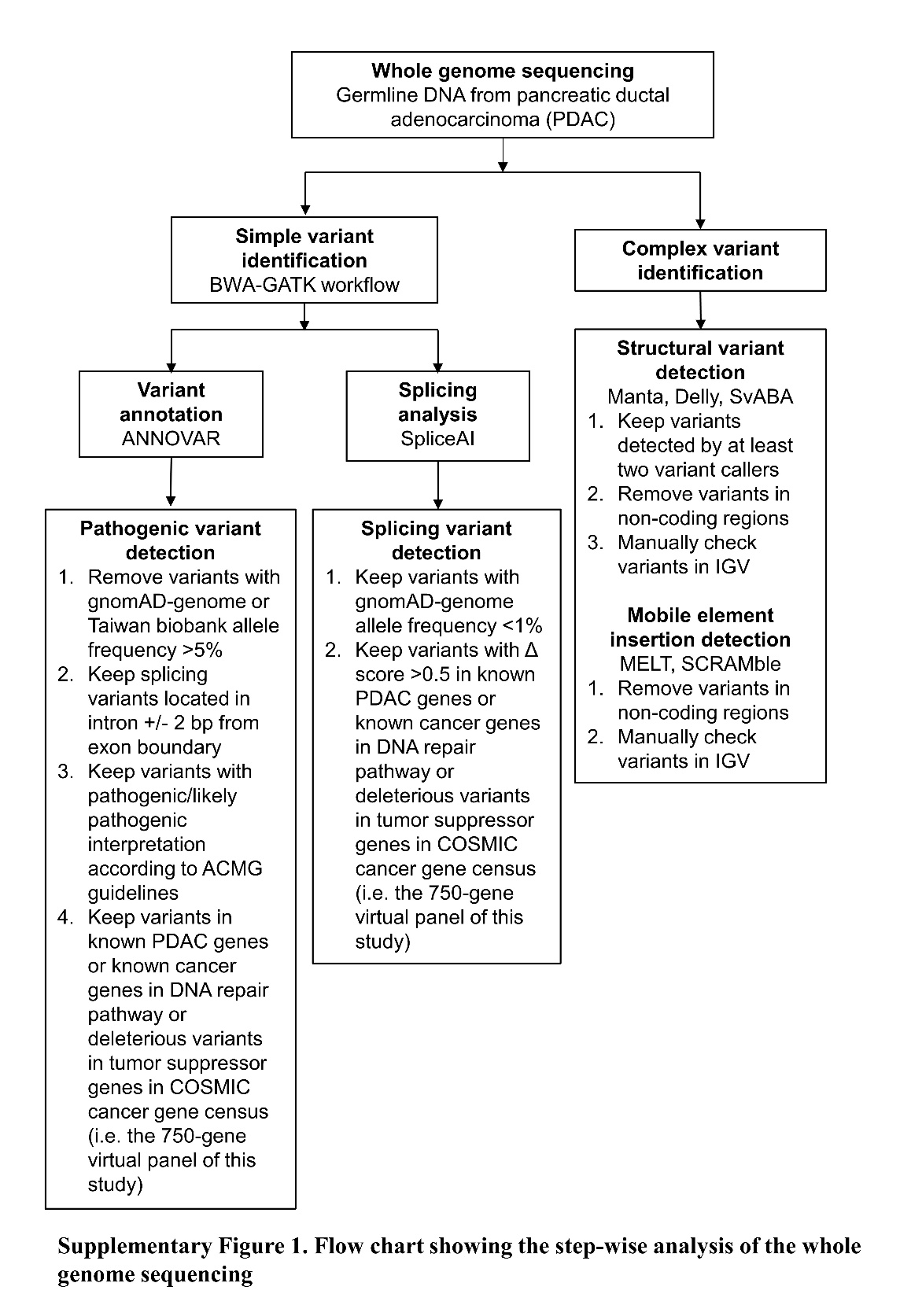

Supplement: Supplementary file 2 [file Table1.DOCX]
